# Lessons to be Learned from Natural Control of HIV – Future Directions, Therapeutic, and Preventive Implications

**DOI:** 10.3389/fimmu.2013.00162

**Published:** 2013-06-25

**Authors:** David Shasha, Bruce D. Walker

**Affiliations:** ^1^The Ragon Institute of MGH, MIT and Harvard, Cambridge, MA, USA

**Keywords:** HIV, elite controllers, CD8^+^ T cells, immune activation, HIV vaccine

## Abstract

Accumulating data generated from persons who naturally control HIV without the need for antiretroviral treatment has led to significant insights into the possible mechanisms of durable control of AIDS virus infection. At the center of this control is the HIV-specific CD8 T cell response, and the basis for this CD8-mediated control is gradually being revealed. Genome wide association studies coupled with HLA sequence data implicate the nature of the HLA-viral peptide interaction as the major genetic factor modulating durable control of HIV, but host genetic factors account for only around 20% of the variability in control. Other factors including specific functional characteristics of the TCR clonotypes generated *in vivo*, targeting of vulnerable regions of the virus that lead to fitness impairing mutations, immune exhaustion, and host restriction factors that limit HIV replication all have been shown to additionally contribute to control. Moreover, emerging data indicate that the CD8^+^ T cell response may be critical for attempts to purge virus infected cells following activation of the latent reservoir, and thus lessons learned from elite controllers (ECs) are likely to impact the eradication agenda. On-going efforts are also needed to understand and address the role of immune activation in disease progression, as it becomes increasingly clear that durable immune control in ECs comes at a cost. Taken together, the research achievements in the attempt to unlock the mechanisms behind natural control of HIV will continue to be an important source of insights and ideas in the continuous search after an effective HIV vaccine, and for the attempts to achieve a sterilizing or functional cure in HIV positive patients with progressive infection.

## Introduction

As the vigorous research of elite controllers (ECs) continue to unveil the various mechanisms behind durable and natural control of HIV, a complex image that includes different factors is exposed. Part of this milieu are components of the adaptive immune system, mainly CD8^+^ and CD4^+^ T cells, innate immune responses and cellular restriction factors, as well as specific characteristics of the infecting virus itself (reviewed in Deeks and Walker, 2007). CD8^+^ T cells were repeatedly found as one of the major contributors to natural control of HIV. Genome wide associations studies consistently recognized the HLA peptide-binding grove (which binds to the CD8^+^ T cell receptor, as well as to receptors on other cells of the immune system) as one of the central genetic associations with HIV disease outcome (Fellay et al., 2007; International HIV Controllers Study et al., 2010). Specific qualities of CD8^+^ T cells from EC were recognized including their ability to inhibit HIV replication *ex vivo* (Sáez-Cirión et al., 2007), to deliver cytotoxic granules to HIV infected target cells (Migueles et al., 2008), to proliferate better (Migueles et al., 2002) and to exhibit multiple effector functions at the same time (“polyfunctionality”) (Betts et al., 2006; Ndhlovu et al., 2012a). It was also demonstrated that CD8^+^ T cells from EC tend to be more cross-reactive (recognizing virus variants and mutants) (Kosmrlj et al., 2010) and to preferentially target more conserved and vulnerable parts of the HIV virus (where the virus pays a higher fitness cost in order to escape from the immune pressure exerted by cytotoxic T cells, CTL; Miura et al., 2009).

It has been shown that CD4^+^ T cells from EC tend to be more polyfunctional, produce more IL-2, and proliferate better (Dyer et al., 2008; Ferre et al., 2010; Vingert et al., 2010). A few studies have also demonstrated that CD4^+^ T cells from EC have reduced permissiveness to HIV infection (Graf et al., 2011; Sáez-Cirión et al., 2011). In that context, it was recently described that cell restriction factors might contribute to the lesser vulnerability of CD4^+^ T cells of EC to HIV infection and replication (Chen et al., 2011). To that we should add the numerous reports that demonstrated important contributions of the innate immune system to elite control including natural killer (Lambotte et al., 2009; Vieillard et al., 2010) and dendritic cells (Huang et al., 2010; Barblu et al., 2012), as well as the contribution of special characteristics of the infecting virus itself (Miura et al., 2010).

Despite the impressive achievements in the field of EC research, our understanding of the mechanisms behind natural control of HIV is still substantially lacking, and the unknown is likely far greater than the known. In this review we examine these achievements in light of their potential contribution to future strategies for prevention and treatment, including vaccine design and attempts for a functional or sterilizing cure. In this way we try to demonstrate that the research of EC goes beyond a pure academic exercise. We emphasize lessons learned from the findings achieved to date and their implications in planning the future steps in the research of this unusual group of patients.

## Lesson I – From Immunology of Elite Controllers to HIV Vaccine Design

A major motivation behind the intensive study of EC is the intention to support the development of an effective HIV vaccine. If we closely examine the insights achieved by the study of EC during the past two decades we can see that several important immune correlates were added to our toolkit to help evaluate vaccine candidates, and some important principles learned can shed light on some of the failures and successes in the field of HIV vaccine research.

In Merck’s phase IIb efficacy (STEP) trial, a replication-defective adenovirus construct (Ad5) containing *Gag*, *Pol*, and *Nef* genes was designed to induce T cell immune responses. The study was prematurely halted as it failed to demonstrate protection from acquisition of infection or to influence post infection viral load (Buchbinder et al., 2008). Indeed, early analysis suggested that the vaccine increased the risk of acquisition. A few principles learned in EC can be demonstrated when examining this important clinical trial. The failure of the vaccine was a surprise, mainly because of positive indications of immunogenicity in earlier phases of the vaccine or similar approaches in non-human primates (Shiver et al., 2002; Priddy et al., 2008), and the understanding that T cells play a central role in natural control of HIV, as demonstrated earlier through CD8^+^ T cell depletion experiments in an animal model of AIDS virus infection (Jin et al., 1999; Schmitz et al., 1999), and by the strong association between certain HLA class I alleles and disease outcome (Fellay et al., 2007; International HIV Controllers Study et al., 2010). The major correlate of protection used to evaluate cell-mediated immune responses in the STEP study was interferon-γ enzyme-linked immunosorbent spot assay (ELISPOT). Such responses were not expected to prevent infection but rather to reduce steady state viremia, but despite induction of these responses, there was no enhanced control of set point viremia following infection (Buchbinder et al., 2008). Interferon-γ-secreting HIV-specific T cells were detected by ELISPOT in the majority of vaccinees, with similar proportion of responders both in those that eventually became infected and those who remained HIV negative (McElrath et al., 2008). Like this vaccine trial, there is no correlation between cellular immune responses measured by ELISPOT assays and clinical outcome (mainly viral load) in several studies looking at EC, yet the clear association of certain HLA alleles with control suggests that CD8^+^ T cell responses play a role (Betts et al., 2001; Addo et al., 2003). The responses measured in the vaccinees in the STEP trial were significantly narrower and of lower magnitude in comparison to responses in EC (McElrath et al., 2008) or in monkeys achieving virologic control after vaccination with adenovirus vector-based SIV vaccine (Barouch et al., 2012). But in that context it should be emphasized that the magnitude and breadth of responses in ELISPOT assays were shown to be a poor predictor of HIV control (Betts et al., 2001), and in fact, it was demonstrated that CD8^+^ T cell responses in EC are usually narrower and more focused on specific epitopes, especially in *Gag* (Migueles et al., 2000). In the STEP trial, on the other hand, CD8^+^ T cell responses were more commonly directed toward *Pol* and *Nef* than toward *Gag* (McElrath et al., 2008). Another major difference between vaccinees in the STEP trial and subjects naturally controlling HIV lies in the way CD8^+^ T cells exhibit their effector functions. While several studies have shown that an effective T cell response in EC is usually polyfunctional (i.e., CD8^+^ T cells are able to produce multiple cytokines simultaneously) (Betts et al., 2006), in the STEP trial most vaccine-induced CD8^+^ T cells produced interferon γ alone or in combination with TNFα only (McElrath et al., 2008).

It’s also worth noticing that in the STEP trial only 41% of the vaccinees developed HIV-specific CD4^+^ T cells that have the potential to support and maintain long-term antiviral CD8^+^ T cell memory, and only 31% mounted both CD4^+^ and CD8^+^ HIV-specific T cells (McElrath et al., 2008). The ability to induce and maintain effective memory is probably one of the central characteristics expected from an effective vaccine. This principle is also important for natural control of HIV, and favorable characteristics of T cell memory have been demonstrated in EC (Ndhlovu et al., 2012b). It has been shown that EC show both effective and functional memory responses, and that central-memory (T_CM_) and effector memory (T_EM_) CD4^+^ T cells in EC are less susceptible to apoptosis and persist longer after repeated stimulations as compared to aviremic HIV infected subjects treated with antiretroviral treatment (van Grevenynghe et al., 2008). Furthermore, it has been recently shown that T_EM_ are the most effective CD8^+^ T cell sub-population at suppressing viral replication in EC (Buckheit et al., 2012). In contrast to states of high antigen load (as is expected in uncontrolled HIV infection), it has been shown that memory cells in states of lower antigen load are able to persist longer, show lower degree of senescence, higher proliferative capacity, and therefore better functional properties (West et al., 2011). Therefore, EC are likely to be an important indicator for the desired way of maintaining memory CD4^+^ and CD8^+^ T cells.

The relevance of these findings to vaccine research was demonstrated in a recent proof-of-concept study in which researchers have used an SIV protein-encoding vector based on rhesus cytomegalovirus (Hansen et al., 2009, 2011). By using a persistent vector that leads to a controlled, continuous level of antigen expression, robust SIV-specific CD4^+^ and CD8^+^ T_EM_ cell responses were primed and maintained. This approach led to impressive protection from acquisition (significantly higher number of challenges needed to cause infection), and importantly to control of post infection viremia in the monkeys, a state that may be similar to elite control or functional cure in humans. The effectiveness of this new approach (in NHP at this stage) renders great hope in the field of HIV vaccine research (Walker et al., 2011). In contrast to older strategies (as that used in the STEP trial) when a non-persistent vector is used and T_CM_ is the predominant memory population elicited, this new strategy leads to development and maintenance of a potent T_EM_ population, thus a faster and more effective systemic and mucosal T cell responses develop. Our own data show that the HIV-specific T_EM_ population in EC predominates and is less prone to apoptosis/deletion as compared to CP (unpublished data).

Although extrapolation of immune correlates of protection in chronic HIV infection to predict protection in vaccine trials might be misleading, the field of HIV vaccine development desperately needs better-defined immune measures that are able to predict vaccine efficacy. The study of ECs, by detailed dissection of the components that lead to natural control, added several such correlates, and it would be reasonable to assume that some of them would be considered in future clinical trials. This includes the ability of CD8^+^ T cells to directly inhibit HIV replication *ex vivo*, their ability to proliferate, and to exhibit polyfunctional effector profile, as well as the composition of the memory subsets induced.

## Lesson II – Elite Controllers, Functional Cure, and HIV Eradication

The most prominent characteristic of EC, complete suppression of the viremia to undetectable levels, can be easily achieved by combination antiretroviral treatment. But some unique and desirable features in EC are absent in non-controllers treated with HAART. Obviously, in contrast to EC, the suppression of the viremia in HAART treated patients requires lifelong medications that are both expensive, and carry the risk for toxicity. Until recently, treatment interruptions universally resulted in viral rebound, and lack of adherence carries the risk for emergence of resistant virus that further complicates the therapeutic regimen. Life expectancy has never been compared between a cohort of untreated EC and non-controllers treated with antiretrovirals, but it has been demonstrated that subjects naturally controlling HIV have smaller HIV reservoirs compared to progressors treated with HAART (Graf et al., 2011; Buzon et al., 2012), and that recovering virus from EC seems to be more difficult as compared to HAART treated patients (Julg et al., 2010). Therefore, it appears as EC are an excellent model of functional cure, a state in which the virus is maximally suppressed, although not cleared, and the integrity of the immune system is maintained relatively intact by the patient’s own defense mechanisms and without the need for life long medications.

Elite controllers inspired researchers to try to induce a similar state of control in infected subjects that do not control the infection spontaneously, and research is on its way in that direction. The most encouraging attempt in that direction was achieved in patients treated with antiretrovirals shortly after infection. In a recent report from France 12 patients out of 75 (16%) who started combination antiretroviral treatment within 10 weeks after HIV infection kept their viral load<50 copies/ml for more than 6 years after discontinuing the antiretroviral treatment (Hocqueloux et al., 2010; Bacchus et al., 2012). The prevalence of elite control in that intervention group was more than 30-fold higher compared to a cohort of non-selected HIV infected individuals (in which the frequency of EC is usually<0.5%). In contrast to EC, the acutely treated cohort was not enriched with protective HLA alleles like HLA-B57 or B27, but rather with HLA-B35, which is associated with rapid disease progression (Carrington et al., 1999), but the reservoir (HIV DNA) in treated patients becoming controllers had similar characteristics compared to EC in terms of magnitude and distribution.

The principle of early treatment was also employed in an interesting case of congenital HIV infection, in which immediate treatment after birth with a full dose antiretroviral regimen led to sustained suppression of viral replication despite prolonged treatment discontinuation (Persaud et al., 2013). These cases of early treatment differ in the approach taken compared to other early intervention studies in both children and adults, in which pulse therapy was largely used, and duration of treatment was much briefer in these patients who did not control after treatment discontinuation (Kaufmann et al., 2004; Fortuny et al., 2011).

It would be important to reveal the mechanisms involved in inducing a state of viral control in patients not harboring protective HLA alleles treated early during the course of acute infection. In a study by our group interesting insights came up from close examination of the transition from acute to chronic controlled infection (Miura et al., 2010). Looking at a cohort of acutely infected subjects not enriched with protective HLA alleles that eventually became HIV controllers significantly higher prevalence of drug resistance or CTL escape mutations associated with impaired viral fitness was found in controllers compared to non-controllers. This work implies that the characteristics of the transmitted virus itself can dictate the fate of the infection natural course. Further examination of the mechanisms leading to elite control after acute infection would broaden our understanding of the way patients become ECs and ways to intervene and induce such a state.

Lessons learned from EC are also influencing cure strategies involving gene therapy. The class I HLA alleles B57 and B27 are the most recognized genetic elements associated with HIV control, and recently several studies have demonstrated that HLA-B27 and B57 restricted TCR clonotypes from EC have superior ability to suppress HIV-1 replication *in vitro* (Chen et al., 2012), greater cross-reactivity to epitope variants (Chen et al., 2012; Ladell et al., 2013) and enhanced loading and delivery of perforin in comparison to HLA-B27 and B57 restricted TCR clonotypes form non-controllers (Chen et al., 2012). Researchers are now examining the possibility to modulate the TCR repertoire of patients not carrying protective HLAs, or having immunodominant non-effective TCR clonotypes by TCR gene transfer (Varela-Rohena et al., 2008; Barsov et al., 2011; Hofmann et al., 2011). By doing that, the purpose is to provide CD8^+^ T cells clones with all the superior characteristics recognized in patients controlling HIV. This idea was proven promising in an animal model at this stage, but active research is still on its way. While adoptive transfer of *ex vivo*-expanded autologous or allogeneic HIV-1-specific CTLs into HIV-1-infected individuals had minimal effect on HIV-1 viral load (Lieberman et al., 1997; Tan et al., 1999; Bolton et al., 2010), as the immunodominant CTL clones in patients with progressive infection are usually exhausted and more susceptible to apoptosis, transduction of primary CD8^+^ T cells with TCR genes from *Gag* specific clone harboring potent inhibition capacity led to an impressive *in vivo* inhibition of HIV in a mouse model (Joseph et al., 2008). This exciting idea is awaiting further evaluation in NHP models and in humans.

The use of genetic engineering in the field of HIV cure research has received inspiration from some other findings in EC and by examining mechanisms involved in viral entry. It is well established that a 32 base pair deletion in the HIV co-receptor C–C chemokine receptor 5 (CCR5) gene confers profound resistance to infection by R5 tropic viruses, and indeed, it has been shown that Δ32 heterozygosity is more prevalent among EC or is associated with lower viral load (Magierowska et al., 1999; International HIV Controllers Study et al., 2010). This important notion not only served the field of antiretroviral therapy development (Gulick et al., 2008), leading to a pharmacologic intervention to block this receptor, but was also employed in an outstanding key work in which HIV cure was achieved by allogeneic bone marrow transplantation in HIV infected patient with acute leukemia. This patient, known as the “Berlin patient,” received hematopoietic stem cell (HSC) graft from a donor homozygous for Δ32 (Hütter et al., 2009) and eventually achieved sustained undetectable viremia despite discontinuation of antiretroviral treatment for more than 3 years (Allers et al., 2011). The same idea is being examined in several studies in which researchers are trying to artificially induce a deletion in the co-receptor by genetic engineering using zinc finger nucleases (ZFN) (Holt et al., 2010). Doing so, researchers were able to disrupt the CCR5 gene in human HSCs and to engraft them into immunodeficient mice. Mice transplanted with ZFN-modified HSC, and later infected with HIV, underwent rapid selection for CCR5-negative cells, and showed significantly lower HIV-1 levels in both peripheral blood and gut mucosa, as well as restoration of normal levels of human CD4^+^ T cell levels throughout their lymphoid tissues. Examination of this concept in humans is currently underway (Tebas et al., 2011; Maier et al., 2013).

Additional work is now taking place examining ways to promote HIV cure, and an international consortium was established specifically for that purpose (International AIDS Society Scientific Working Group on HIV Cure et al., 2012). No doubt that insights learned from the immunology and virology of EC will continue to provide major support to those efforts. The ideas under examination that are inspired by EC research include the efforts to induce strong and effective T cell responses by vaccination [an area that experienced failures and disappointments (Autran et al., 2008), but received some positive results recently utilizing dendritic cells based therapeutic vaccine (García et al., 2013)]. Efforts are also concentrated on attempts to attack and reduce the viral reservoir, to eliminate latently infected cells by activation (using IL-7, histone deacetylase inhibitors, prostratin, and others) in conjunction with induction of effective cytotoxic responses, in addition to the previously mentioned ideas. The possibility of identifying a way for patients to stop antiretroviral treatment and acquire a phenotype similar to ECs is a great challenge that might have significant clinical, epidemiological, and financial implications, but is not without risks due to persistent immune activation. Therefore, scientists and clinicians from different disciplines must continue and work together to take this important goal forward.

## Lesson III – Elite, but Imperfect Control – The Role of Immune Activation

Life expectancy in HIV infected individuals has improved dramatically since the introduction of highly active antiretroviral treatment (Bor et al., 2013), but studies show that it is still compromised compared to matched uninfected controls (Lohse et al., 2007; Nakagawa et al., 2012). Non-AIDS events among HIV infected individuals (including malignancies, cardiovascular, liver, renal, bone disease, and others) are increasingly recognized since HIV became a chronic condition (Guaraldi et al., 2011), and it is clear now that undetectable plasma viremia is not sufficient to prevent such complications. Excess morbidity and mortality in treatment-suppressed HIV infected individuals were attributed to multiple etiologies including toxicity of the antiretroviral treatment, higher rates of co-infections (like HCV and HBV), life style and risk behavior (smoking, drug, and alcohol use), and lower socioeconomic status. But even after adjustment for those factors, higher rates of comorbidities were found compared to uninfected controls, and there is a broad recognition today that a major cause of that is the deleterious effect of the continuous inflammation and immune activation described in these patients. Increased levels of T cell activation (CD38 and HLA-DR), innate immunity activation (CD16 and IL-6 produced by monocytes, IFN-α by dendritic cells, and others) as well as markers of microbial translocation (LPS and other microbial products) and impaired coagulation (d-dimer) were found in chronically infected patients, both treated and untreated, and were associated with increased morbidity (Hsue et al., 2012; Marks et al., 2013; Morse et al., 2013) and mortality (Cozzi-Lepri et al., 2011; Achhra et al., 2012).

In that context, EC serve as an important model to study immune activation. By eliminating confounding factors present in other patient populations (the drug toxicity in HAART treated patients with undetectable viral load, or the influence of the on-going viremia in chronic untreated patients) researchers were able to get a better understanding of the pathogenesis and harm caused by immune activation.

Elevated markers of immune activation and bacterial translocation were found in EC as compared to HAART treated patients (Hunt et al., 2008, 2011), and these abnormalities were associated with lower CD4^+^ cell counts. Excess morbidity from non-AIDS conditions was also found in EC compared to HAART treated patients with undetectable viral load. In a recent work, the prevalence of coronary atherosclerosis (estimated by coronary CT) was unexpectedly higher among EC with sustained viral suppression (no history of viral “blips”) compared to HIV negative individual or HIV infected patients treated with antiretroviral and maintaining undetectable viral load (Pereyra et al., 2012). The higher prevalence of coronary disease was associated with elevated markers of innate immune activation (sCD163, a marker of monocyte activation) as well as markers of CD8^+^ T cells activation (CD38 and HLA-DR). Increased levels of carotid artery intima-media thickness (another manifestation of accelerated atherosclerosis) was also found in EC, and was associated with C reactive protein, a marker of inflammation (Hsue et al., 2009). It has been shown that EC and viremic controllers (those controlling the virus to levels below 2000 copies/ml) have high level of collagen depositions and fibrosis in the gut associated lymphoid tissues, a feature of immune activation and end-organ damage (Sanchez et al., 2013). Surprisingly, the degree of collagen deposition was higher in EC compared to HAART treated patients, uninfected individuals or to that found in the “Berlin patient,” known to be cured of HIV. Some other manifestations of immune dysfunctions were demonstrated in EC. A non-negligible portion (about 10%) of EC progress to AIDS or show progressive loss of their CD4 despite the undetectable viremia (Hunt et al., 2008). Furthermore, in studies in which EC initiated HAART for various clinical indications, CD4^+^ T cell gain was inferior compared to that achieved in non-controllers (Okulicz et al., 2010). These findings imply that despite the impressive control of the plasma viremia in EC, still a significant level of immune activation/dysfunction is present and is associated with various forms of end-organ damage, and might compromise long-term prognosis.

In addition to the observations of increased immune activation, the study of EC helped to explore the various etiologies contributing to its pathogenesis. Like in studies looking at HAART treated patients with undetectable plasma viremia (Buzón et al., 2010), data collected in EC showed that continuous viral replication exists despite the undetectable plasma viremia by standard assays, and it might play a major role in the pathogenesis of chronic immune activation. Utilizing ultrasensitive assays it has been shown that low-level plasma viremia is common in EC, and indeed, 98% of EC had detectable low-level viremia at some point during prolonged follow-up (Hatano et al., 2009). Median plasma viral load of 2 copies/ml was found in EC (Pereyra et al., 2009), a level higher than that usually documented in HAART suppressed patients (Hatano et al., 2009). Cell associated RNA and persistent cell integrated DNA were also evident in EC, and replication competent virus was isolated from these patients (Blankson et al., 2007). Furthermore, some studies have demonstrated that viral evolution occurs in EC, a clear evidence that on-going replication is taking place (O’Connell et al., 2010). These findings imply that the immune system of EC is continuously exposed to low-level release of viral products, which might trigger the state of continuous immune activation and chronic inflammation.

Other factors involved in the pathogenesis of immune activation were described in EC. It is well established that HIV infection is associated with disruption of the gut mucosal barrier and persistent translocation of bacterial products, which in turn lead to systemic immune activation (Brenchley et al., 2006; Wallet et al., 2010). Like in other cohorts of chronically HIV infected individuals, increased markers of microbial translocation were found in EC (Hunt et al., 2008). In a study that compared EC, HIV negative individuals, and untreated HIV infected patients, LPS levels were significantly higher in EC compared to HIV negative controls, and similar to the levels in untreated patients. These findings were associated with higher levels of CD8^+^ T cells activation (Hunt et al., 2008). Such abnormalities might explain the higher levels of fibrosis in the gut associated lymphoid tissue of EC, mentioned before (Sanchez et al., 2013).

To that we should add other conditions that might play a role in the pathogenesis of immune activation in EC, including co-infections with other persistent pathogens like HBV and HCV, the highly effective immune responses in EC that play an important role in controlling the infection but at the same time might facilitate the state of inflammation and immune activation, and other etiologies yet to be defined.

Vigorous research is taking place these days exploring ways to minimize or prevent immune activation and its harmful consequences. The ideas being examined include attempts to eliminate residual viral replication by treatment intensification with antiretrovirals (Buzón et al., 2010; Massanella et al., 2013), interventions to improve gastrointestinal tract integrity (Klatt et al., 2013) and to eliminate other persistent co-infections, treatment with non-specific inhibitors of chronic inflammation like statins, anti-inflammatory agents, anti-cytokine and anti-chemokine agents, immunosuppression, and others (Rajasuriar et al., 2013). The study of EC gives important support in that direction as well, taking advantage of the unique properties of this group of patients. In a recent report, patients naturally controlling HIV (both viremic and ECs) were thoroughly examined after 24 weeks of prospective treatment with combination of three antiretroviral drugs (Hatano et al., 2013). This intervention led to a significant decrease in plasma RNA by ultrasensitive assay, decrease in markers of T cell activation in blood and the gut, and decrease in the percentage of PD-1^+^ CD4^+^ and CD8^+^ T cells. This work demonstrates that significant reduction in immune activation in controllers is possible by eliminating residual viral replication. It would be interesting to examine the influence of other treatment modalities in this group of patients and especially in patients that effectively control HIV but still progress to AIDS.

## Concluding Remarks

The phenomenon of natural control of HIV inspired many researchers who take part in the efforts to develop new preventive and therapeutic modalities for HIV. Exploring the impressive balance between the host and the virus, several new ideas came out that might serve the field of development and evaluation of new vaccine candidates and other modalities of immunotherapy, the field of HIV treatment, and the efforts to eradicate the infection and to lead to HIV cure. The study of EC taught us few lessons about the complex network of components that contribute to the natural control of the virus and the different arms of the immune system involved. The markers of control identified in EC might serve as an important and more accurate correlates of protection in studies of vaccine candidates, and effective immune responses might be transferred by genetic engineering into subjects not naturally controlling HIV. The study of EC also provided new ideas and tools to the field of HIV cure research and to the attempts to achieve HIV eradication, and different ways to induce a state similar to EC are still being examined. The damage caused by chronic inflammation and immune activation is increasingly recognized as an important element compromising the prognosis of HIV infected individuals, and a model of HIV infected patients with undetectable viremia and no exposure to antiretroviral medications is an important tool to study the etiology and influence of these deleterious processes.

As the mechanisms behind natural control of HIV are only partially understood, much effort is still needed to be carried out, and research must continue in new directions and toward new ideas. No doubt the findings revealed by these efforts will have a major impact on the way we treat and prevent HIV in the future.

## Conflict of Interest Statement

The authors declare that the research was conducted in the absence of any commercial or financial relationships that could be construed as a potential conflict of interest.

## References

[B1] Achhra A. C. Amin J. Sabin C. Chu H. Dunn D. Kuller L. H. (2012). Reclassification of risk of death with the knowledge of D-dimer in a cohort of treated HIV-infected individuals. AIDS 26, 1707–1717 10.1097/QAD.0b013e328355d659 22614887 PMC5516536

[B2] Addo M. M. Yu X. G. Rathod A. Cohen D. Eldridge R. L. Strick D. (2003). Comprehensive epitope analysis of human immunodeficiency virus type 1 (HIV-1)-specific T-cell responses directed against the entire expressed HIV-1 genome demonstrate broadly directed responses, but no correlation to viral load. J. Virol. 77, 2081–2092 10.1128/JVI.77.3.2081-2092.2003 12525643 PMC140965

[B3] Allers K. Hutter G. Hofmann J. Loddenkemper C. Rieger K. Thiel E. (2011). Evidence for the cure of HIV infection by CCR5Δ32/Δ32 stem cell transplantation. Blood 117, 2791–2799 10.1182/blood-2010-09-309591 21148083

[B4] Autran B. Murphy R. L. Costagliola D. Tubiana R. Clotet B. Gatell J. (2008). Greater viral rebound and reduced time to resume antiretroviral therapy after therapeutic immunization with the ALVAC-HIV vaccine (vCP1452). AIDS 22, 1313–1322 10.1097/QAD.0b013e3282fdce94 18580611

[B5] Bacchus C. Hocqueloux L. Avettand-Fenoël V. Saez-Cirion A. Mélard A. Descours B. (2012). “Distribution of the HIV reservoir in patients spontaneously controlling HIV infection after treatment interruption,“ in XIX International AIDS Conference (AIDS 2012), abstr. THAA0103, Washington, DC.

[B6] Barblu L. Machmach K. Gras C. Delfraissy J. F. Boufassa F. Leal M. (2012). Plasmacytoid dendritic cells (pDCs) from HIV controllers produce interferon-alpha and differentiate into functional killer pDCs under HIV activation. J. Infect. Dis. 206, 790–801 10.1093/infdis/jis384 22693234

[B7] Barouch D. H. Liu J. Li H. Maxfield L. F. Abbink P. Lynch D. M. (2012). Vaccine protection against acquisition of neutralization-resistant SIV challenges in rhesus monkeys. Nature 482, 89–93 10.1038/nature10766 22217938 PMC3271177

[B8] Barsov E. V. Trivett M. T. Minang J. T. Sun H. Ohlen C. Ott D. E. (2011). Transduction of SIV-specific TCR genes into rhesus macaque CD8+ T cells conveys the ability to suppress SIV replication. PLoS ONE 6:e23703 10.1371/journal.pone.0023703 21886812 PMC3160320

[B9] Betts M. R. Ambrozak D. R. Douek D. C. Bonhoeffer S. Brenchley J. M. Casazza J. P. (2001). Analysis of total human immunodeficiency virus (HIV)-specific CD4(+) and CD8(+) T-cell responses: relationship to viral load in untreated HIV infection. J. Virol. 75, 11983–11991 10.1128/JVI.75.24.11983-11991.2001 11711588 PMC116093

[B10] Betts M. R. Nason M. C. West S. M. De Rosa S. C. Migueles S. A. Abraham J. (2006). HIV nonprogressors preferentially maintain highly functional HIV-specific CD8+ T cells. Blood 107, 4781–4789 10.1182/blood-2005-12-4818 16467198 PMC1895811

[B11] Blankson J. N. Bailey J. R. Thayil S. Yang H. C. Lassen K. Lai J. (2007). Isolation and characterization of replication-competent human immunodeficiency virus type 1 from a subset of elite suppressors. J. Virol. 81, 2508–2518 10.1128/JVI.02165-06 17151109 PMC1865922

[B12] Bolton D. L. Minang J. T. Trivett M. T. Song K. Tuscher J. J. Li Y. (2010). Trafficking, persistence, and activation state of adoptively transferred allogeneic and autologous simian immunodeficiency virus-specific CD8(+) T cell clones during acute and chronic infection of rhesus macaques. J. Immunol. 184, 303–314 10.4049/jimmunol.0902413 19949089 PMC2797565

[B13] Bor J. Herbst A. J. Newell M. L. Barnighausen T. (2013). Increases in adult life expectancy in rural South Africa: valuing the scale-up of HIV treatment. Science 339, 961–965 10.1126/science.1230413 23430655 PMC3860268

[B14] Brenchley J. M. Price D. A. Schacker T. W. Asher T. E. Silvestri G. Rao S. (2006). Microbial translocation is a cause of systemic immune activation in chronic HIV infection. Nat. Med. 12, 1365–1371 10.1038/nm1511 17115046

[B15] Buchbinder S. P. Mehrotra D. V. Duerr A. Fitzgerald D. W. Mogg R. Li D. (2008). Efficacy assessment of a cell-mediated immunity HIV-1 vaccine (the step study): a double-blind, randomised, placebo-controlled, test-of-concept trial. Lancet 372, 1881–1893 10.1016/S0140-6736(08)61591-3 19012954 PMC2721012

[B16] Buckheit R. W. 3rd Salgado M. Silciano R. F. Blankson J. N. (2012). Inhibitory potential of subpopulations of CD8+ T cells in HIV-1-infected elite suppressors. J. Virol. 86, 13679–13688 10.1128/JVI.02439-12 23055552 PMC3503034

[B17] Buzon M. Seiss K. Stone A. Pereyra F. Rosenberg E. Yu X. (2012). “Treatment of early HIV infection reduces viral reservoir to levels found in elite controllers,” in Conference on Retroviruses and Opportunistic Infections (CROI), abstr. 151, Seattle, WA

[B18] Buzón M. J. Massanella M. Llibre J. M. Esteve A. Dahl V. Puertas M. C. (2010). HIV-1 replication and immune dynamics are affected by raltegravir intensification of HAART-suppressed subjects. Nat. Med. 16, 460–465 10.1038/nm.2111 20228817

[B19] Carrington M. Nelson G. W. Martin M. P. Kissner T. Vlahov D. Goedert J. J. (1999). HLA and HIV-1: heterozygote advantage and B∗35-Cw∗04 disadvantage. Science 283, 1748–1752 10.1126/science.283.5408.1748 10073943

[B20] Chen H. Li C. Huang J. Cung T. Seiss K. Beamon J. (2011). CD4+ T cells from elite controllers resist HIV-1 infection by selective upregulation of p21. J. Clin. Invest. 121, 1549–1560 10.1172/JCI44539 21403397 PMC3069774

[B21] Chen H. Ndhlovu Z. M. Liu D. Porter L. C. Fang J. W. Darko S. (2012). TCR clonotypes modulate the protective effect of HLA class I molecules in HIV-1 infection. Nat. Immunol. 13, 691–700 10.1038/ni.2342 22683743 PMC3538851

[B22] Cozzi-Lepri A. French M. A. Baxter J. Okhuysen P. Plana M. Neuhaus J. (2011). Resumption of HIV replication is associated with monocyte/macrophage derived cytokine and chemokine changes: results from a large international clinical trial. AIDS 25, 1207–1217 10.1097/QAD.0b013e3283471f10 21505304 PMC3101710

[B23] Deeks S. G. Walker B. D. (2007). Human immunodeficiency virus controllers: mechanisms of durable virus control in the absence of antiretroviral therapy. Immunity 27, 406–416 10.1016/j.immuni.2007.08.010 17892849

[B24] Dyer W. B. Zaunders J. J. Yuan F. F. Wang B. Learmont J. C. Geczy A. F. (2008). Mechanisms of HIV non-progression; robust and sustained CD4+ T-cell proliferative responses to p24 antigen correlate with control of viraemia and lack of disease progression after long-term transfusion-acquired HIV-1 infection. Retrovirology 5, 112 10.1186/1742-4690-5-112 19077215 PMC2633348

[B25] Fellay J. Shianna K. V. Ge D. Colombo S. Ledergerber B. Weale M. (2007). A whole-genome association study of major determinants for host control of HIV-1. Science 317, 944–947 10.1126/science.1143767 17641165 PMC1991296

[B26] Ferre A. L. Hunt P. W. McConnell D. H. Morris M. M. Garcia J. C. Pollard R. B. (2010). HIV controllers with HLA-DRB1∗13 and HLA-DQB1∗06 alleles have strong, polyfunctional mucosal CD4+ T-cell responses. J. Virol. 84, 11020–11029 10.1128/JVI.00980-10 20719952 PMC2953185

[B27] Fortuny C. Noguera-Julian A. Alsina L. Bellido R. Sánchez E. Muñoz-Almagro C. (2011). Impact of CD4 T cell count on the outcome of planned treatment interruptions in early-treated human immunodeficiency virus-infected children. Pediatr. Infect. Dis. J. 30, 435–438 10.1097/INF.0b013e3181ff8661 21030884

[B28] García F. Climent N. Guardo A. C. Gil C. León A. Autran B. (2013). A dendritic cell-based vaccine elicits T cell responses associated with control of HIV-1 replication. Sci. Transl. Med. 5, 166ra2 10.1126/scitranslmed.3004682 23283367

[B29] Graf E. H. Mexas A. M. Yu J. J. Shaheen F. Liszewski M. K. Di Mascio M. (2011). Elite suppressors harbor low levels of integrated HIV DNA and high levels of 2-LTR circular HIV DNA compared to HIV+ patients on and off HAART. PLoS Pathog. 7:e1001300 10.1371/journal.ppat.1001300 21383972 PMC3044690

[B30] Guaraldi G. Orlando G. Zona S. Menozzi M. Carli F. Garlassi E. (2011). Premature age-related comorbidities among HIV-infected persons compared with the general population. Clin. Infect. Dis. 53, 1120–1126 10.1093/cid/cir627 21998278

[B31] Gulick R. M. Lalezari J. Goodrich J. Clumeck N. DeJesus E. Horban A. (2008). Maraviroc for previously treated patients with R5 HIV-1 infection. N. Engl. J. Med. 359, 1429–1441 10.1056/NEJMoa0803152 18832244 PMC3078519

[B32] Hansen S. G. Ford J. C. Lewis M. S. Ventura A. B. Hughes C. M. Coyne-Johnson L. (2011). Profound early control of highly pathogenic SIV by an effector memory T-cell vaccine. Nature 473, 523–527 10.1038/nature10003 21562493 PMC3102768

[B33] Hansen S. G. Vieville C. Whizin N. Coyne-Johnson L. Siess D. C. Drummond D. D. (2009). Effector memory T cell responses are associated with protection of rhesus monkeys from mucosal simian immunodeficiency virus challenge. Nat. Med. 15, 293–299 10.1038/nm.1935 19219024 PMC2720091

[B34] Hatano H. Delwart E. L. Norris P. J. Lee T. H. Dunn-Williams J. Hunt P. W. (2009). Evidence for persistent low-level viremia in individuals who control human immunodeficiency virus in the absence of antiretroviral therapy. J. Virol. 83, 329–335 10.1128/JVI.01763-08 18945778 PMC2612329

[B35] Hatano H. Yukl S. Ferre A. Sinclair E. Hunt P. Bacchetti P. (2013). “Prospective ART of asymptomatic HIV+ controllers,” in Conference on Retroviruses and Opportunistic Infections (CROI) 2013, abstr. 75LB, Atlanta, GA

[B36] Hocqueloux L. Prazuck T. Avettand-Fenoel V. Lafeuillade A. Cardon B. Viard J. P. (2010). Long-term immunovirologic control following antiretroviral therapy interruption in patients treated at the time of primary HIV-1 infection. AIDS 24, 1598–1601 10.1097/QAD.0b013e32833b61ba 20549847

[B37] Hofmann C. Höfflin S. Hückelhoven A. Bergmann S. Harrer E. Schuler G. (2011). Human T cells expressing two additional receptors (TETARs) specific for HIV-1 recognize both epitopes. Blood 118, 5174–5177 10.1182/blood-2011-04-347005 21926350

[B38] Holt N. Wang J. Kim K. Friedman G. Wang X. Taupin V. (2010). Human hematopoietic stem/progenitor cells modified by zinc-finger nucleases targeted to CCR5 control HIV-1 in vivo. Nat. Biotechnol. 28, 839–847 10.1038/nbt.1663 20601939 PMC3080757

[B39] Hsue P. Y. Hunt P. W. Schnell A. Kalapus S. C. Hoh R. Ganz P. (2009). Role of viral replication, antiretroviral therapy, and immunodeficiency in HIV-associated atherosclerosis. AIDS 23, 1059–1067 10.1097/QAD.0b013e32832b514b 19390417 PMC2691772

[B40] Hsue P. Y. Scherzer R. Hunt P. W. Schnell A. Bolger A. F. Kalapus S. C. (2012). Carotid intima-media thickness progression in HIV-infected adults occurs preferentially at the carotid bifurcation and is predicted by inflammation. J. Am. Heart Assoc. 1, 1–12 10.1161/JAHA.111.000422 23130122 PMC3487373

[B41] Huang J. Burke P. S. Cung T. D. Pereyra F. Toth I. Walker B. D. (2010). Leukocyte immunoglobulin-like receptors maintain unique antigen-presenting properties of circulating myeloid dendritic cells in HIV-1-infected elite controllers. J. Virol. 84, 9463–9471 10.1128/JVI.01009-10 20631139 PMC2937602

[B42] Hunt P. W. Brenchley J. Sinclair E. McCune J. M. Roland M. Page-Shafer K. (2008). Relationship between T cell activation and CD4+ T cell count in HIV-seropositive individuals with undetectable plasma HIV RNA levels in the absence of therapy. J. Infect. Dis. 197, 126–133 10.1086/524143 18171295 PMC3466592

[B43] Hunt P. W. Landay A. L. Sinclair E. Martinson J. A. Hatano H. Emu B. (2011). A low T regulatory cell response may contribute to both viral control and generalized immune activation in HIV controllers. PLoS ONE 6:e15924 10.1371/journal.pone.0015924 21305005 PMC3031543

[B44] Hütter G. Nowak D. Mossner M. Ganepola S. Müssig A. Allers K. (2009). Long-term control of HIV by CCR5 Delta32/Delta32 stem-cell transplantation. N. Engl. J. Med. 360, 692–698 10.1056/NEJMoa0802905 19213682

[B45] International AIDS Society Scientific Working Group on HIV Cure Deeks S. G. Autran B. Berkhout B. Benkirane M. Cairns S. (2012). Towards an HIV cure: a global scientific strategy. Nat. Rev. Immunol. 12, 607–614 10.1038/nri3262 22814509 PMC3595991

[B46] International HIV Controllers Study Pereyra F. Jia X. McLaren P. J. Telenti A. de Bakker P. I. (2010). The major genetic determinants of HIV-1 control affect HLA class I peptide presentation. Science 330, 1551–1557 10.1126/science.1195271 21051598 PMC3235490

[B47] Jin X. Bauer D. E. Tuttleton S. E. Lewin S. Gettie A. Blanchard J. (1999). Dramatic rise in plasma viremia after CD8(+) T cell depletion in simian immunodeficiency virus-infected macaques. J. Exp. Med. 189, 991–998 10.1084/jem.189.6.991 10075982 PMC2193038

[B48] Joseph A. Zheng J. H. Follenzi A. Dilorenzo T. Sango K. Hyman J. (2008). Lentiviral vectors encoding human immunodeficiency virus type 1 (HIV-1)-specific T-cell receptor genes efficiently convert peripheral blood CD8 T lymphocytes into cytotoxic T lymphocytes with potent in vitro and in vivo HIV-1-specific inhibitory activity. J. Virol. 82, 3078–3089 10.1128/JVI.01812-07 18184707 PMC2258988

[B49] Julg B. Pereyra F. Buzón M. J. Piechocka-Trocha A. Clark M. J. Baker B. M. (2010). Infrequent recovery of HIV from but robust exogenous infection of activated CD4(+) T cells in HIV elite controllers. Clin. Infect. Dis. 51, 233–238 10.1086/653677 20550452 PMC3749734

[B50] Kaufmann D. E. Lichterfeld M. Altfeld M. Addo M. M. Johnston M. N. Lee P. K. (2004). Limited durability of viral control following treated acute HIV infection. PLoS Med. 1:e36 10.1371/journal.pmed.0010036 15526059 PMC524377

[B51] Klatt N. R. Canary L. A. Sun X. Vinton C. L. Funderburg N. T. Morcock D. R. (2013). Probiotic/prebiotic supplementation of antiretrovirals improves gastrointestinal immunity in SIV-infected macaques. J. Clin. Invest. 123, 903–907 10.1172/JCI66227 23321668 PMC3561826

[B52] Kosmrlj A. Read E. L. Qi Y. Allen T. M. Altfeld M. Deeks S. G. (2010). Effects of thymic selection of the T-cell repertoire on HLA class I-associated control of HIV infection. Nature 465, 350–354 10.1038/nature08997 20445539 PMC3098720

[B53] Ladell K. Hashimoto M. Iglesias M. C. Wilmann P. G. McLaren J. E. Gras S. (2013). A molecular basis for the control of preimmune escape variants by HIV-specific CD8+ T cells. Immunity 38, 425–436 10.1016/j.immuni.2012.11.021 23521884

[B54] Lambotte O. Ferrari G. Moog C. Yates N. L. Liao H. X. Parks R. J. (2009). Heterogeneous neutralizing antibody and antibody-dependent cell cytotoxicity responses in HIV-1 elite controllers. AIDS 23, 897–906 10.1097/QAD.0b013e328329f97d 19414990 PMC3652655

[B55] Lieberman J. Skolnik P. R. Parkerson G. R. 3rd Fabry J. A. Landry B. Bethel J. Etalname A. (1997). Safety of autologous, ex vivo-expanded human immunodeficiency virus (HIV)-specific cytotoxic T-lymphocyte infusion in HIV-infected patients. Blood 90, 2196–2206 9310470

[B56] Lohse N. Hansen A. B. Pedersen G. Kronborg G. Gerstoft J. Sørensen H. T. (2007). Survival of persons with and without HIV infection in Denmark, 1995-2005. Ann. Intern. Med. 146, 87–95 10.7326/0003-4819-146-2-200701160-00003 17227932

[B57] Magierowska M. Theodorou I. Debré P. Sanson F. Autran B. Rivière Y. (1999). Combined genotypes of CCR5, CCR2, SDF1, and HLA genes can predict the long-term nonprogressor status in human immunodeficiency virus-1-infected individuals. Blood 93, 936–941 9920843

[B58] Maier D. A. Brennan A. L. Jiang S. Binder-Scholl G. K. Lee G. Plesa G. (2013). Efficient clinical scale gene modification via zinc finger nuclease-targeted disruption of the HIV co-receptor CCR5. Hum. Gene Ther. 24, 245–258 10.1089/hum.2012.172 23360514 PMC3609630

[B59] Marks M. A. Rabkin C. S. Engels E. A. Busch E. Kopp W. Rager H. (2013). Markers of microbial translocation and risk of AIDS-related lymphoma. AIDS 27, 469–474 10.1097/QAD.0b013e32835c1333 23169327

[B60] Massanella M. Esteve A. Buzón M. J. Llibre J. M. Puertas M. C. Gatell J. M. (2013). Dynamics of CD8 T-cell activation after discontinuation of HIV treatment intensification. J. Acquir. Immune Defic. Syndr. 63, 152–160 10.1097/QAI.0b013e318289439a 23392458

[B61] McElrath M. J. De Rosa S. C. Moodie Z. Dubey S. Kierstead L. Janes H. (2008). HIV-1 vaccine-induced immunity in the test-of-concept step study: a case-cohort analysis. Lancet 372, 1894–1905 10.1016/S0140-6736(08)61592-5 19012957 PMC2774110

[B62] Migueles S. A. Laborico A. C. Shupert W. L. Sabbaghian M. S. Rabin R. Hallahan C. W. (2002). HIV-specific CD8+ T cell proliferation is coupled to perforin expression and is maintained in nonprogressors. Nat. Immunol. 3, 1061–1068 10.1038/ni845 12368910

[B63] Migueles S. A. Osborne C. M. Royce C. Compton A. A. Joshi R. P. Weeks K. A. (2008). Lytic granule loading of CD8+ T cells is required for HIV-infected cell elimination associated with immune control. Immunity 29, 1009–1021 10.1016/j.immuni.2008.10.010 19062316 PMC2622434

[B64] Migueles S. A. Sabbaghian M. S. Shupert W. L. Bettinotti M. P. Marincola F. M. Martino L. (2000). HLA B∗5701 is highly associated with restriction of virus replication in a subgroup of HIV-infected long term nonprogressors. Proc. Natl. Acad. Sci. U.S.A. 97, 2709–2714 10.1073/pnas.050567397 10694578 PMC15994

[B65] Miura T. Brockman M. A. Schneidewind A. Lobritz M. Pereyra F. Rathod A. (2009). HLA-B57/B∗5801 human immunodeficiency virus type 1 elite controllers select for rare gag variants associated with reduced viral replication capacity and strong cytotoxic T-lymphocyte [corrected] recognition. J. Virol. 83, 2743–2755 10.1128/JVI.00579-09 19116253 PMC2648254

[B66] Miura T. Brumme Z. L. Brockman M. A. Rosato P. Sela J. Brumme C. J. (2010). Impaired replication capacity of acute/early viruses in persons who become HIV controllers. J. Virol. 84, 7581–7591 10.1128/JVI.00286-10 20504921 PMC2897600

[B67] Morse C. G. Dodd L. E. Nghiem K. Costello R. Csako G. Lane H. C. (2013). Elevations in D-dimer and C-reactive protein are associated with the development of osteonecrosis of the hip in HIV-infected adults. AIDS 27, 591–595 10.1097/QAD.0b013e32835c206a 23169328 PMC5507350

[B68] Nakagawa F. Lodwick R. K. Smith C. J. Smith R. Cambiano V. Lundgren J. D. (2012). Projected life expectancy of people with HIV according to timing of diagnosis. AIDS 26, 335–343 10.1097/QAD.0b013e32834dcec9 22089374

[B69] Ndhlovu Z. M. Chibnik L. B. Proudfoot J. Vine S. McMullen A. Cesa K. (2012a). High-dimensional immune monitoring models of HIV-1-specific CD8 T cell responses accurately identify subjects achieving spontaneous viral control. Blood 121, 801–811 10.1182/blood-2012-06-436295 23233659 PMC3563365

[B70] Ndhlovu Z. M. Proudfoot J. Cesa K. Alvino D. M. McMullen A. Vine S. (2012b). Elite controllers with low to absent effector CD8+ T cell responses maintain highly functional, broadly directed central memory responses. J. Virol. 86, 6959–6969 10.1128/JVI.00531-12 22514340 PMC3393560

[B71] O’Connell K. A. Brennan T. P. Bailey J. R. Ray S. C. Siliciano R. F. Blankson J. N. (2010). Control of HIV-1 in elite suppressors despite ongoing replication and evolution in plasma virus. J. Virol. 84, 7018–7028 10.1128/JVI.00548-10 20444904 PMC2898225

[B72] Okulicz J. F. Grandits G. A. Weintrob A. C. Landrum M. L. Ganesan A. Crum-Cianflone N. F. (2010). CD4 T cell count reconstitution in HIV controllers after highly active antiretroviral therapy. Clin. Infect. Dis. 50, 1187–1191 10.1086/651421 20218878

[B73] Pereyra F. Lo J. Triant V. A. Wei J. Buzon M. J. Fitch K. V. (2012). Increased coronary atherosclerosis and immune activation in HIV-1 elite controllers. AIDS 26, 2409–2412 10.1097/QAD.0b013e32835a9950 23032411 PMC3660105

[B74] Pereyra F. Palmer S. Miura T. Block B. L. Wiegand A. Rothchild A. C. (2009). Persistent low-level viremia in HIV-1 elite controllers and relationship to immunologic parameters. J. Infect. Dis. 200, 984–990 10.1086/605446 19656066 PMC3725728

[B75] Persaud D. Gay H. Ziemniak C. Chen Y. H. Piatak M. Chun T.-W. (2013). “Functional HIV cure after very early ART of an infected infant,” in Conference on Retroviruses and Opportunistic Infections, CROI 2013, Atlanta, GA

[B76] Priddy F. H. Brown D. Kublin J. Monahan K. Wright D. P. Lalezari J. (2008). Safety and immunogenicity of a replication-incompetent adenovirus type 5 HIV-1 clade B gag/pol/nef vaccine in healthy adults. Clin. Infect. Dis. 46, 1769–1781 10.1086/587993 18433307

[B77] Rajasuriar R. Khoury G. Kamarulzaman A. French M. A. Cameron P. U. Lewin S. R. (2013). Persistent immune activation in chronic HIV infection: do any interventions work? AIDS 27, 1199–1208 10.1097/QAD.0b013e32835ecb8b PMC4285780 23324661

[B78] Sáez-Cirión A. Hamimi C. Bergamaschi A. David A. Versmisse P. Mélard A. (2011). Restriction of HIV-1 replication in macrophages and CD4+ T cells from HIV controllers. Blood 118, 955–964 10.1182/blood-2010-12-327106 21642597 PMC3148172

[B79] Sáez-Cirión A. Lacabaratz C. Lambotte O. Versmisse P. Urrutia A. Boufassa F. (2007). HIV controllers exhibit potent CD8 T cell capacity to suppress HIV infection ex vivo and peculiar cytotoxic T lymphocyte activation phenotype. Proc. Natl. Acad. Sci. U.S.A. 104, 6776–6781 10.1073/pnas.0611244104 17428922 PMC1851664

[B80] Sanchez J. Hunt P. Jessurun J. Rothenberger M. Reilly C. Jasurda J. (2013). “Persistent abnormalities of lymphoid structures in HIV viremic controllers,” in Conference on Retroviruses and Opportunistic Infections (CROI) 2013, abstr. 74, Atlanta, GA

[B81] Schmitz J. E. Kuroda M. J. Santra S. Sasseville V. G. Simon M. A. Lifton M. A. (1999). Control of viremia in simian immunodeficiency virus infection by CD8+ lymphocytes. Science 283, 857–860 10.1126/science.283.5403.857 9933172

[B82] Shiver J. W. Fu T. M. Chen L. Casimiro D. R. Davies M. E. Evans R. K. (2002). Replication-incompetent adenoviral vaccine vector elicits effective anti-immunodeficiency-virus immunity. Nature 415, 331–335 10.1038/415331a 11797011

[B83] Tan R. Xu X. Ogg G. S. Hansasuta P. Dong T. Rostron T. (1999). Rapid death of adoptively transferred T cells in acquired immunodeficiency syndrome. Blood 93, 1506–1510 10029578

[B84] Tebas P. Levine B. Binder G. Hoxie J. Collman R. Gregory P. (2011). “Disruption of CCR5 in zinc finger nuclease-treated CD4 T cells: phase I trials,” in Conference on Retroviruses and Opportunistic Infections (CROI) 2011, Boston, MA

[B85] van Grevenynghe J. Procopio F. A. He Z. Chomont N. Riou C. Zhang Y. (2008). Transcription factor FOXO3a controls the persistence of memory CD4(+) T cells during HIV infection. Nat. Med. 14, 266–274 10.1038/nm1728 18311149

[B86] Varela-Rohena A. Molloy P. E. Dunn S. M. Li Y. Suhoski M. M. Carroll R. G. (2008). Control of HIV-1 immune escape by CD8 T cells expressing enhanced T-cell receptor. Nat. Med. 14, 1390–1395 10.1038/nm.1779 18997777 PMC3008216

[B87] Vieillard V. Fausther-Bovendo H. Samri A. Debre P. (2010). Specific phenotypic and functional features of natural killer cells from HIV-infected long-term nonprogressors and HIV controllers. J. Acquir. Immune Defic. Syndr. 53, 564–573 10.1097/QAI.0b013e3181d0c5b4 20147841

[B88] Vingert B. Perez-Patrigeon S. Jeannin P. Lambotte O. Boufassa F. Lemaître F. (2010). HIV controller CD4+ T cells respond to minimal amounts of gag antigen due to high TCR avidity. PLoS Pathog. 6:e1000780 10.1371/journal.ppat.1000780 20195518 PMC2829066

[B89] Walker B. D. Ahmed R. Plotkin S. (2011). Moving ahead an HIV vaccine: use both arms to beat HIV. Nat. Med. 17, 1194–1195 10.1038/nm.2529 21988996

[B90] Wallet M. A. Rodriguez C. A. Yin L. Saporta S. Chinratanapisit S. Hou W. (2010). Microbial translocation induces persistent macrophage activation unrelated to HIV-1 levels or T-cell activation following therapy. AIDS 24, 1281–1290 10.1097/QAD.0b013e328339e228 20559035 PMC2888494

[B91] West E. E. Youngblood B. Tan W. G. Jin H. T. Araki K. Alexe G. (2011). Tight regulation of memory CD8(+) T cells limits their effectiveness during sustained high viral load. Immunity 35, 285–298 10.1016/j.immuni.2011.05.017 21856186 PMC3241982

